# Dose Adjustment Helps Obtain Better Outcomes in Multiple Myeloma Patients with Bortezomib, Melphalan, and Prednisolone (VMP) Treatment

**DOI:** 10.4274/tjh.galenos.2019.2019.0306

**Published:** 2019-05-03

**Authors:** Su-Hee Cho, Ho-Jin Shin, Ki Sun Jung, Do Young Kim

**Affiliations:** 1 Pusan National University Yangsan Hospital, Clinic of Hematology-Oncology, Busan, Korea

**Keywords:** Multiple myeloma, Bortezomib, Melphalan, Prednisolone

## Abstract

**Objective:**

Multiple myeloma (MM) has a better survival outcome because of the development of drugs. However, equivalent outcomes cannot be expected from the same drug. Therefore, how the treatment schedule is managed is important. We analyzed VMP (bortezomib, melphalan, and prednisolone) data to determine an effective treatment strategy.

**Materials and Methods:**

We collected the data of 59 patients who were newly diagnosed with MM from January 2012 to April 2017 using electronic medical records. We analyzed baseline characteristics, responses, dose reductions, and survival.

**Results:**

The overall response rate was 86.5% [complete response (CR): 32.2%, very good partial response (VGPR): 37.3%]. The median progression-free survival was 33.6 months and the 5-year overall survival rate was 70%. There were significant better progression-free survival outcomes between CR and non-CR for each of the 4 cycles. Of the four patients who achieved CR after the first cycle, none have had disease progression as of yet. We divided patients into two groups according to the median dose (52.1 mg/m^2^) and we found no differences between the high-dose and low-dose groups. About 78% of patients completed 9-cycle schedules and 84% patients experienced dose reduction, mostly for reasons of non-hematologic toxicities.

**Conclusion:**

Active dose reduction helped to continue treatment and it increased the opportunity to be exposed to drugs. In the end, it resulted in improved outcome.

## Abstract (tr)

**Amaç:**

Günümüzde yeni geliştirilen ilaçlar sayesinde multiple myelom (MM) hastalarında tedavi sonuçları daha iyidir. Ancak, aynı ilaç için her hastada eşit sonuçlar elde edilmesi beklenemez. Bu nedenle, tedavi şemalarının nasıl düzenlendiği büyük önem taşımaktadır. Biz VMP (bortezomib, melfalan ve prednizolon) tedavi verilerini etkin tedavi stratejisi belirlemek amacıyla inceledik.

**Gereç ve Yöntemler:**

Ocak 2012’den Nisan 2017’ye kadar yeni tanı almış 59 MM hastalarının elektronik dosyaları incelendi. Tedavi öncesi özellikler, tedavi cevapları, doz azaltımları ve sağkalımları analiz edildi.

**Bulgular:**

Tüm yanıt oranı %86,5 [tam yanıt (TY) %32,2; çok iyi parsiyel yanıt %37,3) idi. Medyan progresyonsuz yaşam 33,6 ay ve 5 yıllık genel sağkalım %70 idi. Her 4 siklus için de TY sağlanan hastalarda medyan progresyonsuz yaşam TY sağlanamayan hastalara göre istatistiksel olarak anlamlı düzeyde daha iyiydi. İlk siklus sonrasında TY sağlanmış olan 4 hastanın hiçbirinde şu ana kadar progresyon izlenmedi. Hastaları median doza göre (52,1 mg/m^2^) ikiye ayırdığımızda düşük ve yüksek dozda ilaç alanlar arasında fark izlenmedi. Hastaların yaklaşık %78’i planlanan 9 siklusu tamamladı ve %84’ü genellikle hematolojik olmayan toksisiteye bağlı olarak doz azaltılması gerektirdi.

**Sonuç:**

Aktif doz azaltılması, tedavinin devamının sağlanmasına yardım etmekte ve ilaçlara maruziyetinin artmasına fırsat tanımaktadır. Sonuç olarak da daha iyi tedavi sonuçları sağlamaktadır.

## Introduction

Multiple myeloma (MM) has a better survival outcome than other hematologic malignancies such as aggressive lymphoma and acute leukemia [1,2,3]. The survival rate has improved as many new drugs have been developed. Mainstream therapies such as proteasome inhibitors (PIs) and immune modulating drugs (IMiDs), as well as monoclonal antibodies, check point inhibitors, and chimeric antigen receptors, have shown promising results [4,5,6]. However, equivalent outcomes cannot be expected from the same drug. Therefore, how the treatment schedule is managed is as important as the kind of drugs selected. In Korea, government insurance has allowed bortezomib-based treatment as a first-line treatment in transplant-ineligible patients since 2012. We collected and analyzed VMP (bortezomib, melphalan, and prednisolone) data to determine an effective treatment strategy [7,8,9].

## Materials and Methods

### Patients

We enrolled 59 patients who were newly diagnosed with MM and had started VMP therapy from January 2012 to April 2017. All patients were transplant-ineligible. The most common reason for this was age, because the government does not allow transplants over the age of 65 in Korea. Four patients who were under 65 were transplant-ineligible because of poor performance. Data were collected from electronic medical records in two hospitals affiliated with Pusan National University. A response evaluation was conducted in each treatment cycle using serum/urine protein electrophoresis (PEP) and serum free light-chain assay measurements. All patients had bone marrow examinations. A diagnosis was made and a response evaluation was undertaken according to International Myeloma Working Group criteria [10].

### Treatment

All patients received treatment according to the VISTA trial. They received a total of 9 cycles. One cycle was 6 weeks and treatment comprised bortezomib at 1.3 mg/m^2^ on days 1, 4, 8, 11, 22, 25, 29, and 32 in cycles 1-4 and on days 1, 8, 22, and 29 in cycles 5-9; melphalan at 9 mg/m^2^ on days 1-4 in cycles 1-9; and prednisone at 60 mg/m^2^ on days 1-4 in cycles 1-9. Unlike in VISTA, bortezomib was applied subcutaneously.

### Statistical Analysis

Overall survival (OS) and progression-free survival (PFS) were estimated using the Kaplan-Meier method. PFS was calculated from the start-of-treatment date to the date of disease progression, the last follow-up visit, or the date of death if the disease had not progressed until the time of investigation. OS was measured from the date of diagnosis to the date of death, or to the last follow-up visit. Survival rates were compared for statistical differences using log-rank analysis, and we used Pearson’s correlation coefficient for correlation analysis (SPSS 21). A p-value of less than 0.05 was considered to indicate a significant difference.

## Results

### Clinical Characteristics

The median patient age was 72 years (range: 53-81 years), and the male-to-female ratio was 1:1.2. According to the International Scoring System, there were stage I patients (16.9%), stage II patients (30.5%), and stage III patients (52.5%). Of these, only 9 (15.3%) patients had confirmed plasmacytoma at various sites and 11 (18.6%) patients were classified as having light-chain MM (LCMM). Patients in the LCMM category had a high level of only one light-chain without monoclonal immunoglobulin on PEP [11]. Chromosomal abnormalities were identified in 17 (28.8%) patients and complex karyotypes were the most common. Baseline clinical characteristics of all patients are summarized in Table 1.

### Treatment and Response

All patients were treated with VMP according to the schedule in the VISTA trial, with a subcutaneous bortezomib injection [12]. Ten patients (16.9%) received radiotherapy for symptom control before or during the treatment period. A total of 46 patients (78%) completed nine treatment cycles, and 13 patients (22%) stopped treatment early. Disease progression was the main reason for stopping treatment (n=9), followed by death (n=3), and one patient declined to proceed with treatment (n=1). There were 19 (32.2%) patients who achieved complete response (CR), including stringent CR (sCR), while 11 (18.6%) patients achieved very good partial response (VGPR) and 22 (37.3%) patients achieved partial response (PR). The overall response rate (ORR) including sCR, CR, VGPR, and PR was 86.5% (Table 2).

**Figure 1:**
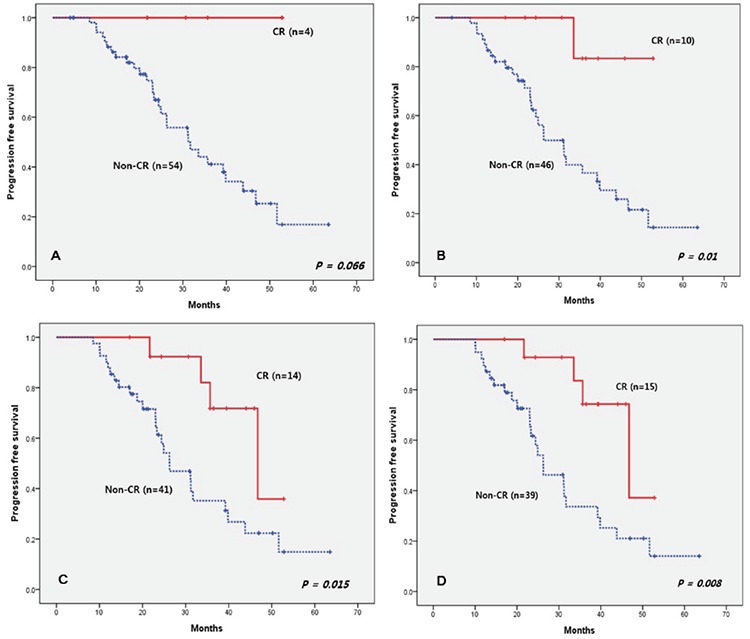
Progression-free survival according to presence of complete response. CR: Complete response.

**Figure 2:**
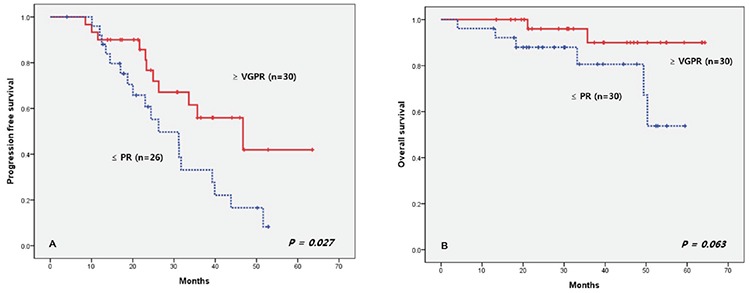
Progression-free survival and overall survival according to very good partial response or partial response. VGPR: Very good partial response, PR: partial response.

The median follow-up duration was 31.1 months (range: 4.0-64.3 months) and the median PFS was 33.6 months (range: 4.0-53.5 months). The median OS was not reached and the 5-year survival rate was 70%. During the follow-up period, 29 patients showed disease progression and most of them received further treatment. In the first cycle, 4 patients achieved CR, and none of them have shown disease progression to date (range: 21.7-52.8 months). In the second, third, and fourth cycles, the patients achieving CR numbered 10, 14, and 15, respectively. All of them showed superior PFS compared to the non-CR group (Figure 1). We also divided the patients into two groups according to VGPR; one group included CR and VGPR while the remaining patients formed the other group for each of the 4 cycles. There was a tendency for a better outcome in the good response group, but this was not statistically significant. In terms of the best response, the ≥VGPR patients showed a statistically significantly improved PFS outcome, with a tendency for an improved OS outcome. The median PFS in the ≥VGPR was 46.7 months, and it was 26.2 months in the ≤PR group (Figure 2).

**Figure 3:**
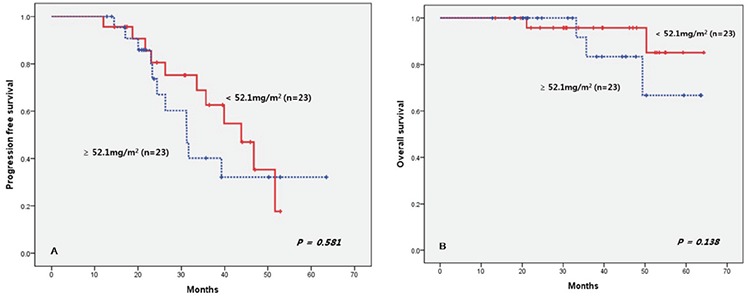
Progression-free survival and overall survival according to median dose.

We divided the patients into groups according to whether they completed 9 cycles of treatment or not. There was significant survival superiority in the 9-cycle group. We only checked the total dose of bortezomib in the patients who completed the 9 treatment cycles. If we included the patients who discontinued treatment, patients with disease progression would be in the low-dose group. Finally, the low-dose group seemed to have poor outcome. Therefore, we selected 46 patients (78%) who had completed 9-cycle schedules and divided them into groups according to the median dose (52.1 mg/m^2^; range: 33.8-67. 5 mg/m^2^). There was no difference in PFS and OS between the high-dose (≥52.1 mg/m^2^) and low-dose (<52.1 mg/m^2^) groups (Figure 3).

### Dose Reduction

During the VMP treatment, 84.7% of patients experienced dose reduction, and 23.7% of patients had the dose reduced twice. One-third of patients (n=16, 32%) experienced dose reduction in the first cycle and 14 patients (28%) and 15 patients (30%) in the second and third cycles, respectively. There was a high dose reduction rate in the first cycle, which showed the tendency of dose reduction. When patients complained about adverse effects, physicians always discussed dose reduction with them. Almost all dose reduction (90%) was done prior to the third cycle, so the proportion of dose reductions had been definitively reduced by the 4^th^ cycle. 

We could check the reason for dose reduction in 32 patients. The main reason for dose reduction was non-hematologic toxicity (92.7%), including peripheral neuropathy (36.6%) (Table 3). Other non-hematologic toxicities were weakness (n=8), emesis (n=4), ileus (n=2), skin rash (n=2), infection (n=2), dizziness (n=2), diarrhea (n=1), mucositis (n=1), and disorientation (n=1).

## Discussion

The VISTA trial reported that a higher dosage improved patient outcome and confirmed the relationship between dose and survival, regardless of discontinuation. The authors of the VISTA report found that patients who had been administered more than 39 mg/m^2^ of bortezomib had better PFS and OS [13]. However, poor responders might have been included in the low-dose group because they analyzed all patients regardless of discontinuation due to poor response. That could make the survival outcome appear worse than the real effect [14]. Because of this, we analyzed only patients who had completed the VMP schedule. The patients who could not finish the whole schedule because of poor response were excluded. In this study, the median dose of bortezomib was 52.1 mg/m^2^, and we found no differences between the high- and low-dose groups in terms of PFS and OS. This showed that the total dose of bortezomib in the same period was not important. 

On the contrary, continuation of treatment can be more effective [15]. In the VISTA trial, 41% of the patients discontinued treatment compared to 22% in our study. The most common reason for discontinuing treatment in the VISTA trial was an adverse event, but no patients stopped treatment because of toxicity in this study. We think that the active dose reduction resulted in low discontinuation because the dose reduction rate (85%) was higher than in the VISTA trial (67%). In particular, if the early dose reduction rate is relatively high, it can also help to continue treatment. Poor disease control may be a concern in early dose reduction. However, there were no differences in survival and response between the dose reduction group and the other groups. We suggest that active dose reduction may help to continue treatment and enable completion of the planned cycles [16,17,18]. It provided patients with an increased opportunity to be exposed to bortezomib and resulted in an improved response. There were more PR patients in our study (88%) than in the VISTA trial (70%). In CR patients (32%), our result was similar to that of VISTA (30%).

The method of bortezomib injection was different compared to VISTA. For this reason, peripheral neuropathy occurred less in this study, and that could be related to lower discontinuation. Nonetheless, the dose reduction rate was higher than in VISTA, which could explain the tendency of active dose reduction strongly.

We think that our results may have been underestimated. In LCMM, many patients did not receive a bone marrow examination, as this is not easy to perform for every response evaluation in practice. Therefore, these patients were usually placed in the PR group, and it is possible that we may have had an even better response if the LCMM patients had received a bone marrow examination.

We also confirmed the importance of an early deep response. All the CR patients in the first cycle have maintained early deep response to date. The CR patients in each cycle between the second and fourth cycles showed a significantly better PFS outcome. In the VISTA trial, the authors also confirmed that CR patients had better PFS than the PR patients [19]. Additionally, we analyzed PFS according to VGPR and confirmed the tendency of a better outcome in the VGPR group. From Korean Multiple Myeloma Working Party data, we know that an early response results in longer survival, and many other studies have also shown similar results [20,21,22].

## Conclusion

We reviewed the records of patients who received VMP treatment, which are similar to previous data. However, we could check that better outcomes were obtained through active dose reduction. The dose reduction makes treatment easier to continue. Finally, it provides an opportunity to administer more drugs and obtain a better response. More data and assessments are required to support these conclusions.

## Figures and Tables

**Table 1:**
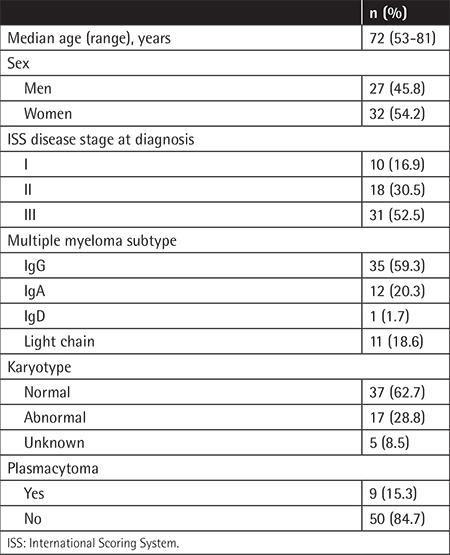
Baseline characteristics (n=59).

**Table 2:**
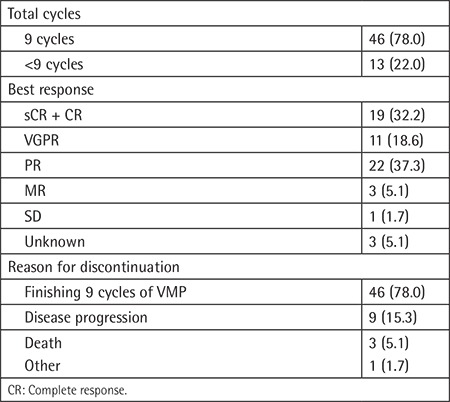
Treatment and response (n=59).

**Table 3:**
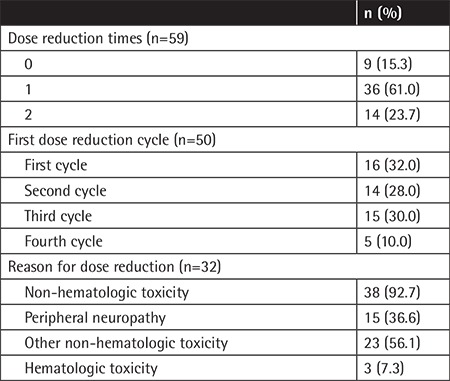
Dose reduction.
